# The impact of fire drills on firefighters’ performance

**DOI:** 10.4102/jamba.v17i2.1825

**Published:** 2025-04-30

**Authors:** Matome R. Ramohale, Botshelo B. Mokaleng, Nthai Monnye

**Affiliations:** 1 Department of Physics, Faculty of Science, Tshwane University of Technology, Pretoria, South Africa; 2 Department of Nuclear Medicine, University of Pretoria, Pretoria, South Africa

**Keywords:** firefighting, fire drills, firefighters’ performance, psychological state, fire management, emergency fire responders, emergency service delivery

## Abstract

Firefighting is one of the most physically demanding, dangerous, and stressful jobs, constituting several physical activities, which include moving equipment up the stairs in tall buildings and deploying charged hoses. In fire management, structured training exercises are fire drills used to prepare staff for successful responses to fires situations. Firefighters, emergency responders, and other pertinent people can practice responding to a real fire by simulating real-life fire events. However, South African fire departments and stations, particularly in Mpumalanga, Nkangala district, are failing to comply with the regulations because they are not conducting these fire drills once a month. This failure to conduct fire drills has resulted in the loss of skills and knowledge. Poor service delivery has become the norm and prevalent. This study aimed to investigate if fire drills can improve the operational efficacy and psychological state of firefighters. The study adopted a quantitative research approach and collected data from randomly sampled participants who work at fire stations located in Nkangala district in Mpumalanga province in South Africa. The Wilcoxon signed-rank statistical test revealed that fire drills do improve operational efficacy and the psychological state of firefighters, demonstrating that fire drills are a key component of fire department’s performance. This study recommends that fire drills must be mandatory for fire departments in South Africa.

**Contribution:**

The study’s findings showed that fire exercises enhance firefighters’ performance and have a favourable effect on their mental health.

## Introduction

Firefighting is one of the most physically demanding, dangerous and stressful jobs, constituting several physical activities, which include moving equipment up the stairs in tall buildings and deploying charged hoses. In fire management, structured training exercises, known as fire drills, are used to prepare staff for effective responses to fire situations. The firefighting profession is one of the most physical, stressful and dangerous occupations in the world (Kales et al. 2003). Fighting fires can be affected by several factors, such as the nature of the emergency response; carrying equipment upstairs in a high-rise; advancing charged hoses, breaking down doors, walls, ceilings and roofs; raising ladders; working overhead with a pike pole or other equipment; rescuing victims; raising and lowering equipment or victims from high-rise windows via ropes; auto extrication and carrying equipment for long distances from the truck to a fire site (Koide 2015). Firefighters may be required to enter a burning building to rescue victims, suppress the blaze and protect property. The inside temperature of a burning structure can be greater than 400°C. However, full personal protective equipment (PPE) allows the firefighters to work amid the heat. Burning structures with high heat causes compromised structural integrity, release of noxious gasses and limitation of use of the senses during an incident response (Koide 2015). Firefighting involves strenuous muscular work while wearing heavy, impermeable, protective clothing, and it is often performed in hot environments. Therefore, strenuous firefighting results in significant heat strain (Romet 1987).

In South Africa, to be a firefighter, you must have passed Grade 12, completed a two- to four-kilometre run and passed a physical ability test (PAT) and a simulation of the firefighting activities (Monnye 2017). Firefighter courses involve basic and advanced competencies, short management learning programmes and diploma courses at the university level from the National Qualifications Framework (NQF) Levels 5 to 10 (Beaton et al. 1997). The basic firefighting courses for professionals are Firefighter I, Firefighter II, Hazardous Material Awareness, Hazmat Operations and First Aid Levels 3–5 (basic fire competencies). Basic competencies as per National Fire Protection Association (NFPA) 472 and 1001 (Standards for hazardous material first responders and for firefighter professional qualifications) comprise more than 200 skills that firefighters should master to be certified. According to [Author(s), in press], firefighters’ training prepares candidates to operate fire equipment, extinguish various kinds of fires, respond to hazardous material incidents, administer first aid and manage a variety of incidents, from non-emergency to emergency calls.

According to Smith and Petruzzello (1998), fire training is divided into two segments: hot or live fire drills and no-fire (dry drills) activities. Hot-fire drills enable a student to deal effectively with incidents involving fires in various occupancies. Dry drills involve simulations aimed at preparing firefighters to effectively use equipment to mitigate any incident safely. Max et al. (2014) assert that drills are either announced or unannounced practice scenarios in real-world settings. Drills usually focus on practising emergency procedures by allowing the observation of behaviour under naturalistic conditions in a specific location. Strategically, fire drills help to build contingency plans by providing an opportunity to question any assumption, deal with potentially difficult situations and evaluate available resources (Wu & Jin 2014). Wu and Jin (2014) expand on available resources, such as smoke-generating machines that may be used to make drills as realistic as possible. According to Gwynne et al. (2017), fire drills should be conducted to ensure that staff understand the emergency fire action plan and its operations. Furthermore, drills enable the fire department to evaluate the effectiveness of the plan and identify any weaknesses in the evacuation strategy. However, the South African NFPA 1043 (Standard for hot fire drills) and NFPA 1410 (Standard on training for initial emergency scene operations) state that a fire department should conduct hot-fire drills once per month.

Among several benefits, the drills improve confidence, heat endurance and prolonged usage of breathing apparatus, prevent injuries, enhance coordination efforts, improve teamwork and enable the fire department to achieve its mission without suffering a loss of life. Fire drills enable a student to deal effectively with incidents involving fires in various occupancies, and therefore fire drills remain the key component of firefighter safety on the job (Hemmatjo et al. 2020; Smith et al. 2024). It is, therefore, mandatory for fire departments to conduct fire drills (Nowack & Niemirowski 2017).

However, it is alarming that many of the fire departments in South Africa, particularly in Nkangala district, Mpumalanga, despite the regulations in place, are not conducting fire drills. Furthermore, according to the Greenbook report by Council for Scientific and Industrial Research CSIR, the likelihood of wildfires under current climatic conditions across the settlements within the Nkangala District Municipality is high (CSIR 2023). This failure to conduct the drills may be attributed to management problems, complacency or laziness. There is, therefore, a need for proper management of fire drills to improve the performance of firefighters. This study provides more information and awareness regarding the importance of conducting regular fire drills. It is therefore important that the level of fire drill awareness among firefighters, such as emergency medical services (EMS) workers, be carefully assessed and documented to aid the design and implementation of appropriate training interventions for fire personnel. Previous studies (Glauberman & Qureshi 2018; Huseyin & Satyen 2006; Lund 2013) conducted in other contexts and countries have demonstrated that studies of this nature are critical as they will assist higher management at fire stations to appreciate the importance of regularly conducting fire drills at their station.

Given the recommendations by SANS and NFPA, South African fire departments and stations are failing to comply with the regulations because they are not conducting these fire drills once a month (Evans & Walls 2024; Raphela & Ndaba 2024). This failure to conduct fire drills has resulted in the loss of skills and knowledge. Poor service delivery has become the norm and prevalent. According to the World Health Organization (WHO) (2023), burning injuries are among major public health problems, particularly in low-income and middle-income countries (such as South Africa), where over 35% of deaths are caused by fire. Globally, fire-related burns alone account for over 300 000 deaths per year, with more deaths from scalds, electricity, chemicals and other forms of burns (South African Department of Cooperate Governance 2020). More than 40% (WHO 2023) of the injuries occur on the ground despite the fact that firefighters spend a very small percentage of their time engaged in fire suppression activities. Firefighters must climb stairs and ladders, carry and use heavy tools (often above their heads or in awkward positions), and they may be called upon to perform difficult rescue operations (Smith 2019).

Firefighters work in dangerous environments. They encounter extreme temperatures, toxic smoke, including carbon monoxide (CO) and hydrogen cyanide (H−C ≡ N) and chaotic conditions that include loud noise and low visibility. Further, this work must be performed with time urgency and is often performed under the psychological stress of knowing that civilians are in imminent danger (Smith 2019). More injuries and fatalities in the line of duty have been recorded because fire departments are not conducting fire drills. This failure to conduct fire drills has caused firefighters to lose their skills and knowledge, leading to poor service delivery and fatalities in the line of duty.

Given the challenges mentioned above that firefighters are facing, there is a need for higher management in the fire department and respective stations to appreciate the importance of fire drills. However, currently, no study that unearths or focusses on investigating the impact of fire drills on firefighters’ performance has been conducted. The main objective of this study was to investigate the impact of fire drills on firefighter performance and their psychological state. To achieve this primary objective, the following sub-objectives were formulated:

- To assess the current state of the fire drills training programme.
- To examine what prevents fire departments from conducting fire drills.
- To investigate the effect of drills on firefighter performance.
- To investigate the effect of drills on firefighters’ psychology.

Based on the main objective, the following hypotheses were formulated:

**H**_**1**_: Fire drills improve firefighters’ performance.

**H**_**0**_ **(null hypothesis):** There is no relationship between fire drills and firefighters’ performance.

**H**_**2**_: Fire drills improve firefighters’ psychological state.

**H**_**0**_ **(null hypothesis):** Fire drills do not improve firefighters’ psychological state.

We anticipate that the findings of this study will theoretically emphasise the strong relationship established between fire drills and firefighters’ performance as well as fire drills and firefighters’ psychological state. Furthermore, we anticipate that the findings of this study can serve as a foundation for future research by scholars wishing to examine the same research problem in other contexts, such as other South African provinces or other nations, either inside or outside of Africa. Practically, higher management at fire stations in the Nkangala district of Mpumalanga can benefit from the study’s conclusions by learning how important fire drills are for enhancing firefighter performance and psychological health.

## Research methods and design

Given that the study intended to collect data from a large number of participants, the study adopted the quantitative approach. All fire stations located in Mpumalanga, Nkangala district consist of 800 firefighters, making the population of the study 800 firefighters. From this population, the sample size was statistically determined to be 260. From the population of 800 participants, 260 participants were randomly sampled to constitute the sample. From the population of 800 firefighters, the study created a single list in an Excel file of all firefighters’ names, surnames and fire stations where they work. The list was alphabetically ordered in ascending order of surnames. From this list, a combination of the random function [=RAND()] and the rank function [=RANK.EQ(C2;$C$2:$C$801)] in Excel were used to assign random numbers to each participant. The random numbers ranged from 1 to 800. The target sample total was 500. Thus, 500 questionnaires were distributed to the identified people within each fire station in Nkangala district in Mpumalanga.

The sampled participants were invited to participate in the study via an online survey questionnaire. The study used an online survey questionnaire because it intended to collect data from a large sample, which was a good representation of the entire population. Furthermore, because the researchers anticipated that some participants would not consent to participating in the study and that some would return an incomplete questionnaire, the researchers opted to increase the sample size to 500. Therefore, 500 questionnaires were distributed, and in return 230 questionnaires were deemed useful. Data for this study were collected in two phases: before and after the fire drills. Data were collected using a closed-ended questionnaire. Data collected before the fire drill were used to gather and assess firefighter perceptions regarding their performance. Then, data collected after the fire drill simulations were collected to assess their perceptions of the impact of fire drills on their performance and psychological state of mind. Both sets of data were used to conduct a comparative analysis to understand the role of fire drills on firefighters’ performance and psychological state of mind. Both data sets were collected from the same 500 participants.

The questionnaire consisted of five sections (A-E). Section A sought to gather the biographical data of the firefighters, which included their academic history and qualifications, their level of rank per department and the number of years served in the fire service. Section B collected data about the daily activities or drills that take place at the fire station. Section C investigated the perception of firefighters on the impact of fire drills on performance. Section D investigated the psychological impact of simulations of fire drills on firefighters. Section E investigated the reasons that prevent firefighters from conducting fire drills. The questions were adopted from already existing validated questions from previous studies (Narciso et al. 2024; Niemann & Thielsch 2020).

The collected data were analysed statistically using descriptive statistics and the Wilcoxon signed-rank test. The ‘Results’ section details and discusses the results obtained.

### Ethical considerations

Ethical clearance to conduct this study was obtained from the Tshwane University of Technology, Faculty Committee for Research Ethics-Science [FCRE-SCI] (No. FCRE 2023/05/004 (SCI) (FCPS 02)).

## Results

According to Pallant (2020), descriptive statistics help in comprehending the distribution of data. The minimum, maximum, mean, standard deviation and skewness are among the statistics that show the central tendency and distribution of the data. The variables whose descriptive statistics were extracted and analysed were those variables that were meant to address the study’s hypothesis. Each variable had descriptive statistics for before and after drills. Table 1 shows the descriptive statistic results obtained from Statistical Package for the Social Sciences (SSPS).

**TABLE 1:** Descriptive statistics for key variables measure pre- and post-drills.

Variable	Statistic				
	Minimum	Maximum	Mean	Standard deviation	Skewness
Pre_Drills_Impact_On_Firefighter Performance	0.00	3.00	1.5435	1.12740	−0.081
Pre_Drills_Effect_On_Psychology	0.00	3.00	1.3130	1.12040	0.279
Post_Drills_Impact_On_Firefighter Performance	0.00	3.00	2.4043	0.64543	−1.015
Post_Drills_Effect_On_Psychology	0.00	3.00	2.0565	0.77139	−0.788
Valid *N* (listwise)	-	-	-	-	-

The variables above were measured using a Likert scale from 0 to 3, with 0 denoting ‘None’ and ‘No Impact’, 1 denoting slight impact, 2 denoting good impact and 3 denoting great impact. According to Table 1, a minimum statistic and maximum statistical value represent the least and maximum option selected. The mean is an average value that takes into account the minimum and maximum values regardless of whether there are outliers. Standard deviation and skewness, according to Pallant (2020), show how scattered the data are from the mean value. According to the study’s findings, when it comes to firefighter performance, a positive skewness indicates that most participants felt that fire drills did not affect firefighters’ abilities. In contrast, a negative skewness indicates that most participants thought fire drills had a positive effect. This skewness also applies to how firefighter psychology is affected by drills.

According to the results in Table 1, all variables before and after the drill had a minimum and maximum value of 0 and 3, respectively. These values mean that some participants, before and after the drills, feel that drills do not impact their performance or their psychology, and some think drills do play an important role in their performance and psychology. Regarding firefighter performance before the drills, the results in Table 1 show that the mean is 1.5435, whereas after the drill, the mean increased to 2.4043. These values indicate that there are clearly some firefighters who appreciate that drills are important for them and that the drills positively impact their performance because there were more participants who selected ‘Fair’ and ‘Good’ after the drills than before the drills. This interpretation also agrees with the skewness value, which shifted from a positive value to a negative value, depicting that the majority of the participants shifted to the right side of the mean.

Regarding the impact on the psychology of firefighters’ performance before the drills, the results in Table 1 show that the mean is 1.3130, whereas after the drill, the mean increased to 2.0565. These values indicate that there are clearly some firefighters who appreciate that drills positively impact their psychology. The implication is that more participants selected ‘Good impact’ and ‘Great impact’ after the drills than before the drills. The appreciation of the impact of drills on firefighters’ psychology also agrees with the skewness value, which shifted from a smaller negative to a bigger negative value, depicting the majority of the participants shifted to the right side of the mean.

These descriptive statistics show that fire drills did impact users as their perceptions changed. However, to know the extent of the impact and if that impact is significant or not, further tests for the hypothesis need to be conducted using advanced inferential statistics such as the Wilcoxon signed-rank test. The hypothesis section presents the Wilcoxon signed-rank test results.

### Hypothesis testing

The non-parametric Wilcoxon signed-rank test was used to test the hypothesis formulated in this study. The non-parametric test was used to test two hypotheses:

**H**_**1**_: Fire drills improve firefighters’ performance.

**H**_**2**_: Fire drills improve firefighters’ psychological state.

Data regarding participants’ perceptions towards their performance and personal psychology were collected before and after fire drill simulations. The intention was to compare their perceptions before and after the drill. This section details the results for the Wilcoxon signed-rank test, which were extracted from SPSS.

#### Hypothesis 1 analysis

The null hypothesis for H_1_ is stated as follows:

**H**_**0**_: There is no relationship between fire drills and firefighters’ performance.

Table 2 shows the overall test of the null hypothesis. The results show that the *p*-value was found to be 0.000, which is less than the significance level of 0.05. Regarding the null hypothesis, this value means that the median of differences between pre-drill perceptions towards firefighters’ performance and post-drill perceptions towards service delivery/firefighters’ performance can be rejected. The null hypothesis states that there is a relationship between fire drills and firefighters’ performance or service delivery. The null hypothesis can also be interpreted as follows: fire drills do not impact or influence service delivery or firefighter performance.

**TABLE 2:** Results of null hypothesis tests for Hypothesis 1.

Hypothesis test summary			
Null hypothesis	Test	Sig.	Decision
The median of differences between Pre_DriIls_lmpact_on_Firefighter Performance and Post_DriIls_lmpact_ On_Firefighter Performance equaIs 0.	Related-SampIes WiIcoxon Signed Rank Test.	0.000	Reject the null hypothesis.

Note: Asymptotic significances are dispIayed. The significance level is 0.05.

Sig., significance.

Because the null hypothesis is rejected, which means that H_1_ is accepted, and this acceptance of H_1_ implies that fire drills do have an influence or impact on service delivery or firefighter performance, and that impact is significant. In order to understand the direction of the impact, the study extracted the descriptive statistics table from the Wilcoxon signed-rank test, and the results are shown in Table 3. According to Table 3, pre-drill perceptions had a mean value of 1.5435, which is lower than that of post-drill perceptions, which had a mean value of 2.4043. This mean value indicates that the overall firefighter performance was improved because the mean of service delivery increased from 1.5435 to 2.4043. Conclusively, the introduction of fire drills positively impacts service delivery.

**TABLE 3:** Descriptive statistics for H_1_ testing.

Variable	*N*	Mean	Standard deviation	Minimum	Maximum
Pre_Drills_Impact_On_Firefighter Performance	230	1.5435	1.12740	0.00	3.00
Post_Drills_Impact_On_Firefighter Performance	230	2.4043	0.64543	0.00	3.00

In order to determine the effect size of this difference, which determines how big the effect or difference is, the researcher extracted the *z*-score test statistics, which are shown in Table 4. The effect size is computed as follows (Equation 1):


$$r=\frac{z}{\sqrt{N}}$$ [Eqn 1]

where *r* is the effect size, *z* is the *z*-score from Table 4 and *N* is the sample size, which is 230 in this study. After computation, the effect size was found to be 0.55, a number that falls in the range of large effects that range from 0.5 to 1.0. Therefore, this implies that the introduction of fire drills significantly improves service delivery.

**TABLE 4:** *Z*-score statistics for H_1_ testing.

Tested Hypothesis	Post_Drills_Impact_On_Firefighter Performance – Pre_Drills_Impact_On_Firefighter Performance
*Z*	−8.270
Asymp. Sig. (2-tailed)	0.000
1. Wilcoxon signed-rank test	
2. Based on negative ranks	

Asymp. Sig., Asymptotic significances; *Z*, score statistics for H_1_ testing.

#### Hypothesis 2 analysis

The null hypothesis for H_2_ is stated as follows:

**H_0_:** Fire drills do not improve firefighters’ psychological state.

Table 5 shows the overall test of the null hypothesis. The results show that the *p*-value was found to be 0.000, which is less than the significance level of 0.05. Regarding the null hypothesis, the median of differences between pre-drill perceptions of psychological effect on firefighters and post-drill perceptions of psychological effect on firefighters can be rejected. The null hypothesis states that fire drills do not improve firefighters’ psychological state.

**TABLE 5:** Results of null hypothesis tests for Hypothesis 2.

Hypothesis test summary			
Null hypothesis	Test	Sig.	Decision
The median of differences between Pre_DriIls_ Effect_On_Psychology and Post_DriIls_Effect_On_Psychology equals 0.	Related-Samples Wilcoxon Signed Rank Test.	0.000	Reject the null hypothesis.

Note: Asymptotic significances are dispIayed. The significance level is 0.05.

Sig., significance.

Because the null hypothesis has been rejected, it means that H_2_ has been accepted. This acceptance implies that fire drills do have an influence or impact on the psychology of firefighters. In order to understand the direction of the impact, the study extracted the descriptive statistics table from the Wilcoxon signed-rank test, and the results are shown in Table 6. According to Table 6, pre-drill perceptions had a mean value of 1.313, which is lower than that of post-drill perceptions, which had a mean value of 2.0565. This value indicates that the overall service delivery level or firefighter performance was improved. Conclusively, the introduction of fire drills positively impacts firefighters’ psychological state.

**TABLE 6:** Descriptive statistics for H_2_ testing.

Variable	*N*	Mean	Standard deviation	Minimum	Maximum
Pre_Drills_Effect_On_Psychology	230	1.3130	1.12040	0.00	3.00
Post_Drills_Effect_On_Psychology	230	2.0565	0.77139	0.00	3.00

In order to determine the effect of the size of this difference, which determines how large the effect or difference is, the researcher extracted the *z*-score test statistics, which are shown in Table 7. After computing the effect size, it was found to be 0.50, which falls in the range of 0.5 to 1.0, indicating a large effect. Therefore, this implies that the introduction of fire drills significantly improved the psychology of firefighters.

**TABLE 7:** *Z*-score statistics for H_2_ testing.

Tested Hypothesis	Post_Drills_Effect_On_Psychology – Pre_Drills_Effect_On_Psychology
*Z*	−7.508
Asymp. Sig. (2-tailed)	0.000
1. Wilcoxon Signed Ranks Test	
2. Based on negative ranks	

Asymp. Sig., Asymptotic significances; *Z*, score statistics for H_2_ testing.

## Discussion

Inferential statistics deduced that fire drills have a significantly large impact on performance and psychological state of mind. This large impact implies that if the department conducts regular fire drills, firefighters will perform better, and their psychological state of mind will be significantly and positively improved. Hypothesis 1 that stipulates that: Fire drills improve firefighters’ performance, was accepted. This confirmation is in line with the recommendation made by Smith (2019) that firefighter fitness programmes should be developed and implemented towards improving health, safety and performance. These results contradict the results obtained by Hemmatjo et al. (2020), who found that cognitive function problems may cause difficulties in work performance. The researchers noted that these cognitive function problems included deterioration of auditory attention, information processing, working memory and visual ability after fire drills. It is important to note that our study did not investigate the impact of fire drills on visual and auditory ability, rather our study measured the perceptions of firefighters regarding their own performance. Future research should then consider conducting a study to measure the performance of firefighters before and after the drills instead of asking them to rate their own perception towards their own performance.

According to the study conducted by Smith (2019), high levels of muscular strength and endurance, anaerobic capacity and aerobic fitness are necessary for fighting fires. However, the data indicate that many firefighters lack these qualities. Several firefighters are overweight and have one or more modifiable risk factors for cardiovascular. Their being overweight may be because of a lack of sufficient fire drills and personal discipline. We believe that this overweight and being out of shape is a significant contributor to the under-performance of fire departments. We also believe that continuous engagement in fire drills will keep firefighters fit and in shape, which will improve their performance and that of the department. According to Smith (2019), firefighters who adhere to well-designed fitness programmes to improve their general health and fitness would benefit both the public and their own health and safety. The lack of high levels of muscular strength and endurance, anaerobic capacity and aerobic fitness aligns with the findings of this research, which demonstrated the value of drills in raising firefighters’ performance levels. Public safety would be better addressed as firefighters perform better. Moreover, the firefighters’ safety will be improved because their enhanced skills and experience will enable them to respond appropriately in various scenarios.

During fire drills, individuals with higher aerobic capacity and fitness were more likely to finish a live firefighting response faster and more effectively than less fit individuals (Petruzzello et al. 2014). This benefit of fitness proves the importance of fire drills as the drills enhance the aerobic capacity of firefighters, which subsequently enhances their ability to finish live tasks quickly. This benefit of fitness supports the finding that regular fire drills improve performance. A study conducted by Dennison et al. (2012) showed that firefighters who regularly train and have greater levels of fitness typically outperform less fit and untrained firefighters at job-specific tasks. Both this study and the study by Dennison et al. (2012) underscore how crucial it is to put in place an exercise programme for firefighters, a direction that the fire departments in Mpumalanga must take.

Regarding Hypothesis 2 that stipulates that fire drills improve firefighters’ psychological state, the hypothesis was accepted. This confirmation means that regular fire drills improve psychological aspects such as morale, confidence, passion and teamwork while reducing psychological aspects such as fear, stress from an accident and feelings of guilt after an accident. The improvement of psychological aspects is in line with what was observed by Petruzzello et al. (2014), which was that after fire drills, significant psychological changes occurred as a result of the firefighting activities. Furthermore, elements that were associated with participants’ psychological discomfort during simplified fire drills found a negative relationship between participants’ psychological discomfort and the frequency of simplified fire drills. This finding implies that, as the frequency of fire drills increases, participants’ psychological discomfort decreases. Firefighters become familiar with the various types of drills and feel comfortable with the drills. Therefore, it is important to do fire drills as often as possible, as suggested by the South African fire department policies. Initially, the firefighters might feel some discomfort with the drills; however, familiarity with the drills decreases their discomfort.

However, this result contradicts the results obtained by Smith et al. (2001), who found that training drills resulted in considerable physiological and psychological strain, which has the potential to impair cognitive function. However, it is important to also note that, even though this researcher’s study and the study by Smith et al. (2001) investigated the same variable (psychology), this study measured psychology in the context of morality, confidence, passion, reduced fear and improved teamwork. In contrast, Smith et al. (2001) investigated psychological exhaustion, which is a different focus.

### Recommendations

The study makes the following recommendations for the legislation that governs the fire brigade service:

- Each fire service should develop a fire drill policy to promote safety drills and to create awareness programmes.
- Fire departments should develop well-maintained structures to record all fire drill performance.
- Fire departments should build long-term collaborative relationships with other departments.
- Finally, appointing qualified and specialised fire drill experts in every fire station can be motivating and will result in the transfer of skills and knowledge to upcoming firefighters.

## Conclusion

This study’s main goal was to investigate how fire exercises affected firefighters’ performance in Nkangala district, Mpumalanga. To accomplish this goal, the study examined the literature on firefighters and fire drills. Information regarding the participants’ opinions regarding fire drills was gathered using an online survey questionnaire. The information was then coded and entered into SPSS for analysis. The data were also subjected to quantitative analysis to evaluate the hypotheses and derive meaning. The study’s findings showed that fire drills enhance firefighters’ performance and have a favourable effect on their mental health.

The study adds a few important theoretical insights. Initially, in the context of Mpumalanga fire departments, the study theoretically contributes to the strong relationship established between fire drills and firefighters’ performance as well as fire drills and firefighters’ psychological state. Moreover, the findings of this study can serve as a foundation for future research by scholars wishing to examine the same research problem in other contexts, such as other South African provinces or other nations, either inside or outside of Africa. Practically, higher management at fire stations in the Nkangala district of Mpumalanga can benefit from the study’s conclusions by learning how important fire drills are for enhancing firefighter performance and psychological health. It is critical to emphasise to upper management the positive impact that regular, ongoing fire drills will have on firefighter satisfaction and service delivery. Based on these findings, management can plan and create strategies and training programmes for fire drills that will improve the delivery of firefighting services and the lives of firefighters.

The study has some limitations that can shape future research. Participants’ perceptions of the effect of fire drills on their performance were measured. As a result, performance was evaluated according to participant perception. There may be a limit to this measurement. An alternative method of measuring performance would be for the researcher to devise a way of measuring performance changes independently before and after fire drills. In the researcher’s opinion, this method of measuring performance is more reliable than the one employed in this study. Furthermore, methods allow participants to share their thoughts on the impact of fire drills on their performance. Future research should repeat the same study with other provinces and then compare the results obtained in this study, thereby validating the results of this study.
